# Supplementary material for: Stacking Model‐Based Classifiers for Dealing With Multiple Sets of Noisy Labels
Source: Biom J. 2025 Mar 12;67(2):e70042. doi: 10.1002/bimj.70042 (PMC11898607; doi:10.1002/bimj.70042)
Supplement: Supplementary file 1 — Supporting Information [file BIMJ-67-e70042-s001.zip › density-based-ensemble-model-main/competitors_real_data/netlab-master/netlab-master/help/confmat.htm]

Netlab Reference Manual confmat


# confmat

## Purpose

Compute a confusion matrix.

## Synopsis

```
[C, rate] = confmat(y, t)
```

## Description

`[C, rate] = confmat(y, t)` computes the confusion matrix `C`
and classification performance `rate` for the predictions mat{y}
compared with the targets `t`. The data is assumed to be in a
1-of-N encoding, unless there is just one column, when it is assumed to
be a 2 class problem with a 0-1 encoding. Each row of `y` and `t`
corresponds to a single example.

In the confusion matrix, the rows represent the true classes and the
columns the predicted classes. The vector `rate` has two entries:
the percentage of correct classifications and the total number of
correct classifications.

## See Also

`conffig`, `demtrain`

---

**Pages:**
Index


---

Copyright (c) Ian T Nabney (1996-9)
